# Functional mapping imprinted quantitative trait loci underlying developmental characteristics

**DOI:** 10.1186/1742-4682-5-6

**Published:** 2008-03-17

**Authors:** Yuehua Cui, Shaoyu Li, Gengxin Li

**Affiliations:** 1 Department of Statistics & Probability, Michigan State University, East Lansing, MI 48824, USA

## Abstract

**Background:**

Genomic imprinting, a phenomenon referring to nonequivalent expression of alleles depending on their parental origins, has been widely observed in nature. It has been shown recently that the epigenetic modification of an imprinted gene can be detected through a genetic mapping approach. Such an approach is developed based on traditional quantitative trait loci (QTL) mapping focusing on single trait analysis. Recent studies have shown that most imprinted genes in mammals play an important role in controlling embryonic growth and post-natal development. For a developmental character such as growth, current approach is less efficient in dissecting the dynamic genetic effect of imprinted genes during individual ontology.

**Results:**

Functional mapping has been emerging as a powerful framework for mapping quantitative trait loci underlying complex traits showing developmental characteristics. To understand the genetic architecture of dynamic imprinted traits, we propose a mapping strategy by integrating the functional mapping approach with genomic imprinting. We demonstrate the approach through mapping imprinted QTL controlling growth trajectories in an inbred F_2 _population. The statistical behavior of the approach is shown through simulation studies, in which the parameters can be estimated with reasonable precision under different simulation scenarios. The utility of the approach is illustrated through real data analysis in an F_2 _family derived from LG/J and SM/J mouse stains. Three maternally imprinted QTLs are identified as regulating the growth trajectory of mouse body weight.

**Conclusion:**

The functional iQTL mapping approach developed here provides a quantitative and testable framework for assessing the interplay between imprinted genes and a developmental process, and will have important implications for elucidating the genetic architecture of imprinted traits.

## Background

Hunting for genes underlying mendelian disorders or quantitative traits has been a long-term effort in genetical research. Most current statistical approaches to gene mapping assume that the maternally and paternally derived copies of a gene in diploid organisms have a comparable level of expression. This, however, is not necessarily true as revealed by recent studies, in which some genes show asymmetric expression, and their expression in the offspring depends on the parental origin of their alleles [1-3]. This phenomenon, termed genomic imprinting, results from the modification of DNA structure rather than changes in the underlying DNA sequences. As one type of epigenetic phenomenon, genomic imprinting has greatly shaped modern research in genetics since its discovery. Some previously puzzling genetic phenomena can now be explained by imprinting theory. However, little is known about the size, location and functional mechanism of imprinted genes in development.

The selective control of gene imprinting is unique to placental mammals and flowering plants. There is increasing evidence that many economically important traits and human diseases are influenced by genomic imprinting [3-6]. More recent studies have shown that genomic imprinting might be even more common than previously thought [7]. Despite its importance, the study of genomic imprinting is still in its early infancy. The biological function of genomic imprinting in shaping an organism's development is still unclear. Recent publications have shown that the majority of imprinted genes in mammals play an important role in controlling embryonic growth and development [8,9], and some involve in post-natal development, affecting suckling and metabolism [9,10]. The malfunction of imprinted genes at any developmental stage could lead to substantially abnormal characters such as cancers or other genetic disorders. It is therefore of paramount importance to identify imprinted genes and to understand at which developmental stage they function, to help us explore opportunities to prevent, control and treat diseases therapeutically. With the development of new biotechnology coupled with computationally efficient statistical tools, it is now possible to map imprinted genes and understand their roles in disease susceptibility.

Several studies have shown that the effects of imprinted quantitative trait loci (iQTL) can be estimated and tested in controlled crosses of inbred or outbred lines [6,11-15]. These approaches are designed on the traditional QTL mapping framework where a phenotypic trait is measured at certain developmental stage for a mapping subject, ignoring the dynamic features of gene expression. As a highly complex process, genomic imprinting involves a number of growth axes operating coordinately at different development stages [16]. Changes in gene expression at different developmental stages reflect the dynamic changes of gene function over time. They also reflect the response of an organism to either internal or external stimuli, so it can redirect its developmental trajectory to adapt better to environmental conditions, and thereby to increase its fitness [17]. For this reason, incorporating such information into genetic mapping should provide more information about the genetic architecture of a dynamic developmental trait.

When a developmental feature of an imprinted trait is considered, traditional iQTL mapping approaches that only consider the phenotypic trait measured at a particular time point will be inappropriate for such an analysis. In fact, for a quantitative trait of developmental behavior, the genetic effect at time *t *(denoted as *G*_*t*_) is composed of the genetic effect at time *t *- 1 (denoted as *G*_*t*-1_) and the extra genetic effect from time *t *- 1 to *t *(denoted as *G*_Δ*t*_) [18]. Therefore, the phenotypic trait measured at time *t *reflects the cumulative gene effects from initial time to *t*, and is highly correlated with the trait measured at time *t *- 1. The correlations among traits measured at different time periods (i.e., different developmental stages) thus provide correlation information about gene expressions, and hence tell us how genes mediate to respond to internal and external stimuli. Current imprinting QTL (iQTL) mapping approaches, by ignoring the correlations among traits measured at different developmental stages, could therefore potentially overestimate the number and the effective size of iQTLs, and lead to wrong inferences.

Although conditional QTL analysis can reduce bias and increase detecting power by partitioning the genetic effect in a conditional manner [18], analysis of traits at each measurement time point is still less powerful and less attractive than analysis by considering measurements at different developmental stages jointly [19]. The recent development of functional mapping brings challenges as well as opportunities for mapping genes responsible for dynamic features of a quantitative trait [17,19,20]. Functional mapping is the integration between genetic mapping and biological principles through mathematical equations. The relative merits of functional mapping in biology lie in the strong biological relevance of QTL detection, and its statistical advantages are that it reduces data dimensions and increases the power and stability of QTL detection. By incorporating various mathematical functions into the mapping framework, functional mapping has great flexibility for mapping genes that underlie complex dynamic/longitudinal traits. It provides a quantitative framework for assessing the interplay between genetic function and developmental pattern and form.

In this article, we extend our previous work of interval iQTL mapping to functional iQTL mapping by incorporating biologically meaningful mathematical functions into a QTL mapping framework. We illustrate the idea through an inbred line F_2 _design, although it can be easily extended to other genetic designs. To distinguish the genetic differences between the two reciprocal heterozygous forms derived from an F_2 _population, information about sex-specific differences in the recombination fraction is used. Monte Carlo simulations are performed to evaluate the model performance under different scenarios considering the effect of sample size, heritability and imprinting mechanism. A real example is illustrated in which three iQTLs affecting the growth trajectory of body weight in an F_2 _family derived from two different mouse strains are identified through a genome-wide linkage scan.

## Methods

### Functional QTL Mapping

Statistical methods for mapping QTL underlying developmental characteristics such as growth or HIV dynamics have been developed previously [19,20]. The so called functional mapping approach has been recently applied to mapping QTL underlying programmed cell death [21,22]. Functional mapping is derived under the finite mixture model-based likelihood framework. In the mixture model, each observation *y *is modelled as a mixture of *J *(known and finite) components. The distribution for each component corresponds to the genotype category depending on the underlying genetic design. For an F_2 _design, there are three mixture components (*J *= 3). The density function for each genotype component is assumed to follow a parametric distribution (*f*) such as Gaussian, which can be expressed as:

$$y\sim p(y|\vec{\pi },\vec{\phi },\eta )=\pi_{1}f_{1}(y;\phi_{1},\eta )+\pi_{2}f_{2}(y;\phi_{2},\eta )+\pi_{3}f_{3}(y;\phi_{3},\eta )$$

where $\vec{\pi }$ = (*π*_1_, *π*_2_, *π*_3_) is a vector of mixture proportions which are constrained to be non-negative and sum to unity; $\vec{\phi }$ = (*ϕ*_1_, *ϕ*_2_, *ϕ*_3_) is a vector for the component specific parameters, with *ϕ*_*j *_being specific to component *j*; and *η *contains parameters (i.e., residual variance) that are common to all components.

For an F_2 _design initiated with two contrasting homozygous inbred lines, there are three genotypes at each locus. Suppose there is a putative segregating QTL with alleles *Q *and *q *that affects a developmental trait such as growth. In a QTL mapping study, the QTL genotype is generally considered as missing, but can be inferred from the two flanking markers. The missing QTL genotype probability *π*_*j *_can be calculated as the conditional probability of the QTL genotype given the observed flanking marker genotypes. For a population with structured pedigree like an F_2 _population, it can be expressed in terms of the recombination fractions, whereas for a natural population, it can be expressed as a function of linkage disequilibria. The derivations of the conditional probabilities of QTL genotypes can be found in the general QTL mapping literature [23].

In functional mapping, the parameters $\vec{\phi }$ = (*ϕ*_1_, *ϕ*_2_, *ϕ*_3_) specify the underlying developmental mean function (**m**). For an F_2 _design, there are three sets of mean functions corresponding to three QTL genotypes. To reduce the number of parameters and enhance the interpretability of functional mapping, the mean process is modelled by certain biologically meaningful mathematical functions, either parametrically or nonparametrically. Suppose that the phenotypic traits are acquired from *n *individuals, and that *t *measurements are made on each individual *i*. Let the response of individual *i *at time *t *be denoted by *y*_*i*_(*t*), *i *= 1, ⋯, *n*; *t *= 1, ⋯, *τ*. Then the response can be modelled as

*y*_*i*_(*t*) = *f *(*t*) + *e*_*i*_(*t*),

where *f*(*t*) is a linear or nonlinear function evaluated at time *t*, depending on the underlying developmental pattern; *e*_*i*_(*t*) is the residual error, which is assumed to be normal with mean zero and variance *σ*^2^(*t*). The intra-individual correlation is specified as *ρ*, which leads to the covariance for individual *i *at two different time points, *t*_1 _and *t*_2_, expressed as cov(*y*_*i*_(*t*_1_), *y*_*i*_(*t*_2_)) = $\rho \sigma_{t_{1}}\sigma_{t_{2}}$. Assuming multivariate normal distribution, the density function for each progeny *i *who carries genotype *j *can be expressed as

$$f_{j}(y_{i}|\Omega_{q},\Omega_{r})=\frac{1}{{\left(2\pi \right)}^{\tau /2}{\left|\Sigma \right|}^{1/2}}\exp\left[-\frac{1}{2}(y_{i}-m_{j})\Sigma^{-1}{\left(y_{i}-m_{j}\right)}^{T}\right],$$

where **m**_*j *_= [*m*_*j*_(1), ⋯, *m*_*j*_(*τ*)] is the mean vector common for all individuals with genotype *j*, which can be evaluated through function *f *in Model (2). The unknown parameters that specify the position of QTL within a marker interval are arrayed in **Ω**_*r*_. The parameters that define the mean and the covariance functions are arrayed in **Ω**_*q*_.

Since we do not observe the QTL genotype, the distribution of *y *is modelled through a finite mixture model given in Model (1). At a particular time point (say *t*), the genetic effect can be obtained by solving the following equations

$$a(t)=\frac{1}{2}(m_{1}(t)-m_{3}(t))\text{ and }d(t)=m_{2}(t)-\frac{1}{2}(m_{1}(t)+m_{3}(t))$$

where *a*(*t*) and *d*(*t*) are the additive and dominant effects at time *t*, respectively.

### Functional iQTL Mapping

#### Modelling the imprinted mean function

In an F_2 _population, three QTL genotypes are segregated. The three QTL genotypes may have different expressions which result in three different mean trajectories. Considering the imprinting property of an iQTL, we introduce the notation for the parental origin of alleles inherited from both parents. Let *Q*_*M *_and *q*_*M *_be two alleles inherited from the maternal parent, and *Q*_*P *_and *q*_*P *_be two alleles derived from the paternal parent. The subscripts *M *and *P *refer to maternal and paternal origin, respectively. These four parentally specific alleles form four distinct genotypes expressed as *Q*_*M*_*Q*_*P*_, *Q*_*M*_*q*_*P*_, *q*_*M*_*Q*_*P*_, and *q*_*M*_*q*_*P*_. In contrast, in a regular QTL mapping study without distinguishing the allelic parental origin, the two reciprocal heterozygotes, *Q*_*M*_*q*_*P *_and *q*_*M*_*Q*_*P*_, are collapsed to one heterozygote. When a QTL is imprinted, the four QTL genotypes show different gene expressions, which result in different developmental growth trajectories. For a maternally (or paternally) imprinted QTL, the allele inherited from the maternal (or paternal) parent is not expressed. Thus, two growth trajectories would be expected. By testing the differences of the four growth trajectories, one can test whether there is a QTL, and whether the QTL is imprinted.

For simplicity, we use numerical notation to denote the four parent-of-origin-specific genotypes, i.e., *Q*_*M*_*Q*_*P *_= 1, *Q*_*M*_*q*_*P *_= 2, *q*_*M*_*Q*_*P *_= 3, and *q*_*M*_*q*_*P *_= 4. The mean functions of these genotypes are denoted as **m**_*j*_, (*j *= 1, ⋯, 4). We know that for an imprinted gene, the expression of an allele depends on its parental origin. On a developmental scale, the two reciprocal heterozygotes, *Q*_*M*_*q*_*P *_and *q*_*M*_*Q*_*P*_, may present different mean trajectories. The degree of imprinting of an iQTL can thus be assessed by the genotype-specific parameters. Through testing the difference between the mean functions of the two reciprocal heterozygotes, we can assess the imprinting property of a QTL. An overlap of the two trajectories for the two reciprocal heterozygotes indicates no sign of imprinting.

For a developmental characteristic such as growth, it is well known that the underlying trajectory can be described by a universal growth law, which follows a logistic growth function [24]. At a developmental stage, say time *t*, the mean value of an individual carrying QTL genotype j can be expressed by

$$m_{j}(t)=\frac{\alpha_{j}}{1+\beta_{j}e^{-\gamma_{j}t}},$$

where the growth parameters (*α*_*j*_, *β*_*j*_, *γ*_*j*_) describe asymptotic growth, initial growth and relative growth rate, respectively [25]. With estimated growth parameters, we can easily retrieve the genotypic means at every time point by simply plugging *t *into Equation (5). This modelling approach can significantly reduce the number of unknown parameters to be estimated, especially when the number of measurement points is large [19].

At a particular time point (say *t*), the mean expression of an individual carrying QTL genotype *j *can be evaluated through the three growth parameters (*α*_*j*_, *β*_*j*_, *γ*_*j*_). On the basis of the univariate imprinting model given in [12], we can partition the genetic effects at time *t *as the allele-specific effects, i.e.

$$\begin{array}{c} a_{M}(t)=\frac{1}{2}(m_{1}(t)+m_{2}(t)-m_{3}(t)-m_{4}(t))\\ a_{P}(t)=\frac{1}{2}(m_{1}(t)-m_{2}(t)+m_{3}(t)-m_{4}(t))\\ d(t)=m_{1}(t)-m_{2}(t)-m_{3}(t)+m_{4}(t) \end{array}$$

where *a*_*M *_and *a*_*P *_refer to the additive effects of alleles inherited from mother and father, respectively; *d *refers to the allele dominant effect.

To illustrate the idea, we use the growth trait to demonstrate the mapping principle. The idea can be easily extended to other developmental characteristics. For developmental characteristics other than growth, different mathematical functions should be developed. Some flexible choices include nonparametric regressions based on smoothing splines or orthogonal polynomials [21].

#### Modelling the covariance structure

To understand how QTL mediate growth, it is essential to take correlations among repeated measures into account [19]. The repeated measures provide correlation information on gene expression. Hence, dissection of the intra-individual correlation will help us to understand better how genes function over time. One commonly used model for covariance structure modelling is the first-order autoregressive (AR(1)) model [26], expressed as

*σ*^2^(1) = ⋯ = *σ*^2^(*τ*) = *σ*^2^

for the variance, and

$$\sigma (t_{k},t_{k^{\prime }})=\sigma^{2}\rho^{\left|t_{k^{\prime }}-t_{k}\right|}(t_{k^{\prime }}>t_{k})$$

for the covariance between any two time points *t*_k _and *t*_k'_, where 0 <*ρ *< 1 is the proportion parameter with which the correlation decays with time lag.

For a developmental characteristic such as growth, the inter-individual variation generally increases as time increases, which leads to a nonstantionary variance function. Since the AR(1) covariance model assumes stationary variance, it can not be applied directly. To stabilize the variance at different measurement time points, we apply a multivariate Box-Cox transformation to stabilize the variance [27], which has the form

$$z_{i}(t)=\{\begin{array}{lll} \frac{y_{i}{\left(t\right)}^{\lambda (t)}-1}{\lambda (t)}, & \text{if} & \lambda \neq 0\\ \log(y_{i}(t)), & \text{if} & \lambda =0 \end{array}$$

The Box-Cox transformation ensures the homoscedasticity and normality of the response *y*. For repeated measures or longitudinal studies, a reasonable constraint is to set *λ*(*t*) = *λ *for all *t*. Then the optimal choice of *λ *can be estimated from the data. To preserve the interpretability of the estimated mean parameters, Carroll and Ruppert [28] proposed a transform-both-sides (TBS) model in which the same transformation form is applied to both sides of Model (2). For a log-transformation, this results in log*y*_*i*_(*t*) = log*f*(*t*) + *e*_*i*_(*t*). Wu et al. [29] later showed the favorable property of this approach in functional mapping. For the modelling purpose of stabilizing variances, we simply adopt the log-transformation in the current setting.

Alternatively, one can model the covariance structure nonstationarily without transforming the original data. Among a pool of choices, the structured antedependence (SAD) model [30] displays a number of favorable merits. The SAD model of order p for modelling the error term in Eq. (2) is given by

*e*_*i*_(*t*) = *φ*_1_*e*_*i*_(*t *- 1) + ⋯ + *φ*_*p*_*e*_*i*_(*t *- *r*) + *ε*_*i*_(*t*)

where *ε*_*i*_(*t*) is the "innovation" term assumed to be independent and distributed as $N(0,\sigma_{t}^{2})$. Therefore, the variance-covariance matrix can be expressed as

**Σ **= **AΣ**_*ε*_**A**^T^,

where **Σ**_*ε *_is a diagonal matrix with diagonal elements being the innovation variance; **A **is a lower triangular matrix which contains the antedependence coefficient *φ*_*r*_. The SAD order (*p*) can be selected through an information criterion [31]. The SAD(*r*) model has been previously applied in functional mapping of programmed cell death [21].

### Parameter Estimation

Assuming inter-individual independence, the joint likelihood function is given by

$$L(\Omega |z,ℳ)=\prod_{i=1}^{n}\sum_{j=1}^{4}\pi_{j|i}f_{j}(z_{i}|\Omega ,ℳ)$$

where **z**_*i *_= [*z*_*i*_(1), ⋯, *z*_*i*_(*τ*)] is the observed log-transformed trait vector for individual *i *(*i *= 1, ⋯, *n*) over *τ *time points; *f*_*j *_is the multivariate normal density function with log-transformed mean for QTL genotype *j*; *π*_*j*|*i *_(*j *= 1, ⋯, 4) is the mixture proportion for individual *i *with genotype *j*, which is derived assuming a sex-specific difference in recombination rate and can be found in [12]. The unknown parameters in **Ω **comprise three sets, one defining the co-segregation between the QTL and markers and thereby the location of the QTL relative to the markers, denoted by **Ω**_*r*_, and the other defining the distribution of a growth trait for each QTL genotype, denoted by **Ω**_*q *_= (**Ω**_*m*_, **Ω**_*v*_), where $\Omega_{m}=(\Omega_{m_{1}},\Omega_{m_{2}},\Omega_{m_{3}},\Omega_{m_{4}})$ defines the mean vector for different genotypes and **Ω**_*v *_defines the covariance parameters.

We implement the EM algorithm to obtain the maximum likelihood estimates (MLEs) of the unknown parameters. The first derivative of the log-likelihood function, with respect to specific parameter *ϕ *contained in **Ω**, is given by

$$\begin{array}{ll} & \frac{\partial }{\partial \Omega_{\phi }}\log\ell (\Omega |z,ℳ)\\ = & \sum_{i=1}^{n}\sum_{j=1}^{4}\frac{\pi_{j|i}\frac{\partial }{\partial \Omega_{\phi }}f_{j}(z_{i}|\Omega ,ℳ)}{\sum_{j^{\prime }=1}^{4}\pi_{j^{\prime }|i}f_{j^{\prime }}(z_{i}|\Omega ,ℳ)}\\ = & \sum_{i=1}^{n}\sum_{j=1}^{4}\frac{\pi_{j|i}f_{j}(z_{i}|\Omega ,ℳ)}{\sum_{j^{\prime }=1}^{4}\pi_{j^{\prime }|i}f_{j^{\prime }}(z_{i}|\Omega ,ℳ)}\frac{\partial }{\partial \Omega_{\phi }}\log f_{j}(z_{i}|\Omega ,ℳ)\\ = & \sum_{i=1}^{n}\sum_{j=1}^{4}\Pi_{j|i}\frac{\partial }{\partial \Omega_{\phi }}\log f_{j}(z_{i}|\Omega ,ℳ) \end{array}$$

where we define

$$\Pi_{j|i}=\frac{\pi_{j|i}f_{j}(z_{i}|\Omega ,ℳ)}{\sum_{j^{\prime }=1}^{4}\pi_{j^{\prime }|i}f_{j^{\prime }}(z_{i}|\Omega ,ℳ)}$$

The MLEs of the parameters contained in (**Ω**_*m*_, **Ω**_*v*_) are obtained by solving

$$\frac{\partial }{\partial \Omega_{\phi }}\log\ell (\Omega |z,ℳ)=0$$

Direct estimation is unavailable since there is no closed form for the MLEs of parameters. The EM algorithm is applied to solve these unknowns iteratively.

**E-step**: Given initial values for (**Ω**_*m*_, **Ω**_*v*_), calculate the posterior probability matrix **Π **= {Π_*j*|*i*_} in Eq. (8).

**M-step**: With the updated posterior probability **Π**, we can update the parameters contained in **Ω**_*q*_. The maximization can be implemented through an iteration procedure or through the Newton-Raphson or other algorithm such as simplex algorithm [32].

The above procedures are iteratively repeated between (8) and (9), until a certain convergence criterion is met. For details of the EM algorithm, one can refer to [19]. The converged values are the MLEs of the parameters. The initial values under the alternative hypothesis are generally set as the estimated values under the null. Note also that in the above algorithm, we do not directly estimate the QTL-segregating parameters (**Ω**_*r*_). In general, we use a grid search approach to estimate the QTL location by searching for a putative QTL at every 1 or 2 cM on a map interval bracketed by two markers throughout the entire linkage map. The log-likelihood ratio test statistic for a QTL at a testing position is displayed graphically to generate a log-likelihood ratio plot called LR profile plot. The genomic position corresponding to a peak of the profile is the MLE of the QTL location.

We have found that the algorithm is sensitive to initial values, particularly the mean values of the two reciprocal heterozygotes. To make sure the parameters are converged to the "correct" ones, we normally give different initial values for the two reciprocal heterozygotes and check which one produces the highest likelihood value. The ones which produce higher likelihood value are considered as the MLEs.

### Hypothesis Testing

#### Global QTL test

Testing whether there is a QTL affecting the developmental trajectory is the first step toward understanding of genetic architecture of an imprinted trait. Once the MLEs of the parameters are obtained, the existence of a QTL affecting the growth curve can be tested by formulating the following hypotheses

$$\{\begin{array}{lll} {\text{H}}_{0} & : & \Omega_{m_{1}}\equiv \cdots ,\equiv \Omega_{m_{4}}\\ {\text{H}}_{1} & : & \text{The equalities above do not hold}, \end{array}$$

where H_0 _corresponds to the reduced model, in which the data can be fit by a single curve, and H_1 _corresponds to the full model, in which there exist different curves to fit the data. The above test is equivalent to test

$$\{\begin{array}{lll} {\text{H}}_{0} & : & \alpha_{1}=\alpha_{2}=\alpha_{3}=\alpha_{4},\beta_{1}=\beta_{2}=\beta_{3}=\beta_{4},\gamma_{1}=\gamma_{2}=\gamma_{3}=\gamma_{4}\\ {\text{H}}_{1} & : & \text{The equalities above do not hold}, \end{array}$$

The statistic for testing the hypotheses is calculated as the log-likelihood (LR) ratio of the reduced to the full model

$$\text{LR}=-2[\log L(\tilde{\Omega }|z,ℳ)-\log L(\bar{\Omega }|z,ℳ)]$$

where $\tilde{\Omega }$ and $\bar{\Omega }$ denote the MLEs of the unknown parameters under H_0 _and H_1_, respectively. An empirical approach to determining the critical threshold is based on permutation tests [33].

#### Imprinting test

Rejection of the null hypothesis in Test (10) at a particular genomic position indicates evidence of a QTL at that locus. Next, we would like to know the imprinting property of a detected QTL. To test if a detected QTL is imprinted or not, we develop the following hypothesis

$$\{\begin{array}{lll} {\text{H}}_{0} & : & \alpha_{2}=\alpha_{3},\beta_{2}=\beta_{3},\gamma_{2}=\gamma_{3}\\ {\text{H}}_{1} & : & \text{The equalities above do not hold}, \end{array}$$

The null hypothesis states that the two reciprocal QTL genotypes have the same mean curve and hence have the same gene expression, i.e., the expressions of genotypes *Q*_*M*_*q*_*P *_and *q*_*M*_*Q*_*P *_are independent of allelic origin. Rejection of the null hypothesis indicates evidence of genomic imprinting.

Following Test (11), if the null is rejected, further tests can be done to test whether an iQTL is maternally imprinted or paternally imprinted. The following hypothesis tests can be formulated

$$\{\begin{array}{lll} {\text{H}}_{0} & : & \alpha_{1}=\alpha_{2},\beta_{1}=\beta_{2},\gamma_{1}=\gamma_{2}\\ {\text{H}}_{1} & : & \text{The equalities above do not hold}, \end{array}$$

for testing paternally imprinted QTL and

$$\{\begin{array}{lll} {\text{H}}_{0} & : & \alpha_{1}=\alpha_{3},\beta_{1}=\beta_{3},\gamma_{1}=\gamma_{3}\\ {\text{H}}_{1} & : & \text{The equalities above do not hold}, \end{array}$$

for testing maternally imprinted QTL.

The null hypothesis in Test (12) states that the two QTL genotypes *Q*_*M*_*Q*_*P *_and *Q*_*M*_*q*_*P *_have the same mean curves and hence same expressions (i.e., allele inherited from the paternal parent does not express).

The iQTL identified can then be claimed as a paternally imprinted QTL. Similarly, if one fails to reject the null in Test (13), the conclusion that there is maternal imprinting can be reached.

Note that the imprinting test (11) is only conducted at the position where a significant QTL is declared on the basis of Test (10). So Test (11) is a point test. Tests (12) and (13) are only conducted when the null in Test (11) is rejected. We can either use the likelihood ratio test or a nonparametric test based on the area under the curve (AUC). The idea of the AUC test is that if two genotypes have the same expression, the area under the developmental curve would be the same. The AUC for QTL genotype j is defined as

$${\text{AUC}}_{j}{|}_{1}^{\tau }=\int_{1}^{\tau }\frac{\alpha_{j}}{1+\beta_{j}e^{-\gamma_{j}t}}dt=\frac{\alpha_{j}}{\gamma_{j}}\log(\frac{\beta_{j}+e^{\tau \gamma_{j}}}{\beta_{j}+e^{\gamma_{j}}})$$

Similarly, Tests (11)–(13) can be defined accordingly based on the AUC. For example, to test (12), the hypothesis would be simplified to

$$\{\begin{array}{lll} {\text{H}}_{0} & : & AUC_{1}{|}_{1}^{\tau }=AUC_{2}{|}_{1}^{\tau }\\ {\text{H}}_{1} & : & AUC_{1}{|}_{1}^{\tau }\neq AUC_{2}{|}_{1}^{\tau } \end{array}$$

The significance of Tests (11)–(13) can be evaluated on the basis of permutations. In our simulation study, we found that the test based on the AUC is more sensitive and powerful than the one based on the likelihood ratio test.

#### Regional test

Even though a mean curve can be modelled throughout a continuous function, genes may not function across all the observed stages. For imprinted genes, loss of imprinting (LOI) is reported in the literature [34]. The question of how a QTL exerts its effects on an interval across a growth trajectory (say [*t*_1_, *t*_2_]) can be tested using a regional test approach based on the AUC. The AUC for genotype *j *at a given time interval is calculated as

$${\text{AUC}}_{j}=\int_{t_{1}}^{t_{2}}\frac{\alpha_{j}}{1+\beta_{j}e^{-\gamma_{j}t}}dt$$

If the AUCs of the four genotypes for a testing period [*t*_1_, *t*_2_] are the same, we claim there is no QTL effect at that time interval. The hypothesis test for the genetic effect over a period of growth can be formulated as

$$\{\begin{array}{lll} {\text{H}}_{0} & : & {\text{AUC}}_{1}{|}_{t_{1}}^{t_{2}}=\cdots ={\text{AUC}}_{4}{|}_{t_{1}}^{t_{2}}\\ {\text{H}}_{1} & : & \text{The equalities above do not hold} \end{array}$$

This test can detect if a QTL exerts an early gene effect or triggers a late effect.

## Results

### Monte Carlo Simulation

Monte Carlo simulations are performed to evaluate the statistical behavior of the developed approach. Consider an F_2 _population initiated with two contrasting inbred lines, with which a 100 *cM *long linkage group composed of 6 equidistant markers is constructed. A putative QTL that affects the imprinted growth process is located at 46 cM from the first marker on the linkage group. The marker genotypes in the F_2 _family are simulated by mimicking sex-specific recombination fractions in mice, i.e., *r*_*M *_= 1.25*r*_*P *_. The Haldane map function is used to convert the map distance into the recombination fraction. Data are simulated with different specifications, namely different heritability levels (H^2 ^= 0.1 vs 0.4) and different sample sizes (*n *= 200 vs 500). For each F_2 _progeny, its phenotype is simulated with 10 equally spaced time points. The covariance structure is simulated assuming the first-order AR(1) model. Note that the variance parameter (*σ*^2^) is calculated on the basis of the log-transformed data.

Several data sets are simulated assuming no imprinting, partial imprinting, complete maternal and paternal imprinting. The simulation results are summarized in Tables 1, 2, 3, 4. As we expected, the precision of parameter estimates is increasing with the increase of the sample size and heritability under different imprinting scenarios. For example, when a QTL is not imprinted (Table 4), the RMSE of the parameter *a *for genotype *Q*_*M*_*Q*_*P *_decreases from 0.397 to 0.327, an 18% increase in precision when the sample size increases from 200 to 500 with fixed heritability level (0.1). For the same parameter, when a QTL is completely maternally imprinted, a reduction in RMSE from 0.478 to 0.305 is observed (Table 1). When we fix the sample size and increase the heritability level, the reduction in RMSE is even more noteworthy. For example, under fixed sample size (*n *= 200), the RMSE of the parameter a for QTL genotype *Q*_*M*_*Q*_*P *_is reduced from 0.397 to 0.137, a 65% increase in precision compared to an 18% increase when sample size increases from 200 to 500 with fixed heritability (Table 4). Large heritability infers high genetic variability and low environmental variation [35]. Therefore, to increase the precision of parameter estimation, well managed experiments in which environmental variation is reduced is more important than just simply increasing sample sizes.

**Table 1:** The MLEs of the model parameters and the QTL position derived from 200 simulation replicates assuming complete maternal imprinting. The square root of the mean square errors (RMSEs) of the MLEs are given in parentheses.

			*Q*_*M*_*Q*_*P*_			*Q*_*M*_*q*_*P*_			*q*_*M*_*Q*_*P*_			*q*_*M*_*q*_*P*_			Residual	
							
*H*^2^	*n*	Position (cM)	*α*_1_ 36.5	*β*_1_ 6.5	*γ*_1_ 0.75	*α*_2_ 33.5	*β*_2_ 5.5	*γ*_2_ 0.75	*α*_3_ 36.5	*β*_3_ 6.5	*γ*_3_ 0.75	*α*_4_ 33.5	*β*_4_ 5.5	*γ*_4_ 0.75	*σ*^2^	*ρ* 0.8
0.1	200	45.31 (7.506)	36.52 (0.478)	6.50 (0.135)	0.75 (0.010)	33.74 (0.992)	5.60 (0.333)	0.75 (0.014)	36.22 (0.998)	6.38 (0.341)	0.75 (0.015)	33.47 (0.438)	5.50 (0.117)	0.75 (0.011)	0.0086 (0.001)	0.79 (0.014)
	NI	45.54 (8.267)	36.5685 (0.515)	6.53 (0.147)	0.75 (0.010)	35.03 (1.579)	5.99 (0.504)	0.75 (0.007)	35.03 (1.554)	5.99 (0.523)	0.75 (0.007)	33.42 (0.464)	5.48 (0.115)	0.75 (0.011)	0.009 (0.001)	0.81 (0.013)
0.1	500	45.88 (3.615)	36.54 (0.305)	6.51 (0.087)	0.75 (0.006)	33.72 (0.866)	5.57 (0.285)	0.75 (0.009)	36.32 (0.832)	6.42 (0.272)	0.75 (0.009)	33.49 (0.289)	5.50 (0.071)	0.75 (0.006)	0.0089 (0.0005)	0.80 (0.008)
	NI	46.12 (4.483)	36.59 (0.323)	6.53 (0.089)	0.75 (0.006)	34.97 (1.486)	5.97 (0.477)	0.75 (0.005)	34.97 (1.541)	5.97 (0.501)	0.75 (0.005)	33.41 (0.304)	5.48 (0.079)	0.75 (0.006)	0.009 (0.0006)	0.81 (0.01)
0.4	200	46.33 (2.671)	36.51 (0.206)	6.50 (0.057)	0.75 (0.004)	33.54 (0.352)	5.51 (0.111)	0.75 (0.004)	36.46 (0.361)	6.49 (0.112)	0.75 (0.004)	33.49 (0.186)	5.50 (0.045)	0.75 (0.004)	0.0015 (0.0003)	0.80 (0.014)
	NI	47.63 (4.625)	36.67 (0.251)	6.56 (0.080)	0.75 (0.004)	34.96 (1.502)	5.98 (0.495)	0.75 (0.005)	34.96 (1.582)	5.98 (0.532)	0.75 (0.004)	33.38 (0.212)	5.45 (0.065)	0.75 (0.004)	0.0018 (0.0003)	0.82 (0.027)
0.4	500	46.07 (1.684)	36.48 (0.119)	6.50 (0.031)	0.75 (0.002)	33.50 (0.127)	5.50 (0.034)	0.75 (0.003)	36.49 (0.145)	6.50 (0.037)	0.75 (0.003)	33.49 (0.123)	5.50 (0.030)	0.75 (0.002)	0.0015 (0.0001)	0.80 (0.007)
	NI	48.21 (2.644)	36.67 (0.215)	6.56 (0.072)	0.75 (0.002)	34.97 (1.487)	5.98 (0.486)	0.75 (0.002)	34.97 (1.551)	5.98 (0.526)	0.75 (0.002)	33.36 (0.182)	5.45 (0.057)	0.75 (0.003)	0.0018 (0.0003)	0.83 (0.029)

The location of the simulated QTL is described by the map distances (in cM) from the first marker of the linkage group (100 cM long). The hypothesized *σ*^2 ^value is 0.009 for *H*^2 ^= 0.10 and 0.0015 for *H*^2 ^= 0.4. The analysis results by non-imprinting model are indicated by "NI".

**Table 2:** The MLEs of the model parameters and the QTL position derived from 200 simulation replicates assuming complete paternal imprinting. The square root of the mean square errors (RMSEs) of the MLEs are given in parentheses.

			*Q*_*M*_*Q*_*P*_			*Q*_*M*_*q*_*P*_			*q*_*M*_*Q*_*P*_			*q*_*M*_*q*_*P*_			Residual	
							
*H*^2^	*n*	Position (cM)	*α*_1_ 36.5	*β*_1_ 6.5	*γ*_1_ 0.75	*α*_2_ 36.5	*β*_2_ 6.5	*γ*_2_ 0.75	*α*_3_ 33.5	*β*_3_ 5.5	*γ*_3_ 0.75	*α*_4_ 33.5	*β*_4_ 5.5	*γ*_4_ 0.75	*σ*^2^	*ρ * 0.8
0.1	200	43.56 (8.519)	36.84 (0.567)	6.54 (0.141)	0.75 (0.010)	36.84 (0.974)	6.51 (0.297)	0.75 (0.016)	33.89 (0.904)	5.61 (0.293)	0.75 (0.017)	33.87 (0.585)	5.57 (0.140)	0.75 (0.012)	0.01 (0.0012)	0.81 (0.019)
0.1	500	45.27 (4.683)	36.90 (0.508)	6.56 (0.107)	0.75 (0.006)	36.88 (0.731)	6.55 (0.204)	0.75 (0.010)	33.87 (0.732)	5.56 (0.210)	0.75 (0.011)	33.82 (0.437)	5.55 (0.090)	0.75 (0.007)	0.009 (0.0005)	0.81 (0.011)
0.4	200	45.67 (3.217)	36.51 (0.193)	6.50 (0.050)	0.75 (0.003)	36.47 (0.374)	6.49 (0.114)	0.75 (0.004)	33.52 (0.348)	5.51 (0.108)	0.75 (0.004)	33.52 (0.168)	5.51 (0.046)	0.75 (0.004)	0.0015 (0.0004)	0.80 (0.012)
0.4	500	46.04 (1.579)	36.48 (0.118)	6.50 (0.035)	0.75 (0.002)	36.49 (0.129)	6.50 (0.039)	0.75 (0.002)	33.50 (0.121)	5.50 (0.033)	0.75 (0.003)	33.50 (0.111)	5.50 (0.028)	0.75 (0.003)	0.0015 (0.0001)	0.80 (0.008)

The location of the simulated QTL is described by the map distances (in cM) from the first marker of the linkage group (100 cM long). The hypothesized *σ*^2 ^value is 0.009 for *H*^2 ^= 0.10 and 0.0015 for *H*^2 ^= 0.4.

**Table 3:** The MLEs of the model parameters and the QTL position derived from 200 simulation replicates assuming partial imprinting. The square root of the mean square errors (RMSEs) of the MLEs are given in parentheses.

			*Q*_*M*_*Q*_*P*_			*Q*_*M*_*q*_*P*_			*q*_*M*_*Q*_*P*_			*q*_*M*_*q*_*P*_			Residual	
							
*H*^2^	*n*	Position (cM)	*α*_1_ 36.5	*β*_1_ 6.5	*γ*_1_ 0.7	*α*_2_ 35.5	*β*_2_ 6.5	*γ*_2_ 0.7	*α*_3_ 34.5	*β*_3_ 6	*γ*_3_ 0.7	*α*_4_ 33.5	*β*_4_ 5.5	*γ*_4_ 0.7	*σ*^2^	*ρ* 0.8
0.1	200	45.37 (3.932)	36.51 (0.324)	6.49 (0.096)	0.70 (0.006)	35.18 (0.891)	6.27 (0.363)	0.70 (0.011)	34.85 (0.863)	6.23 (0.356)	0.70 (0.012)	33.51 (0.316)	5.51 (0.084)	0.70 (0.007)	0.0043 (0.001)	0.79 (0.014)
0.1	500	45.96 (2.206)	36.52 (0.225)	6.50 (0.060)	0.70 (0.004)	35.15 (0.686)	6.30 (0.321)	0.70 (0.008)	34.88 (0.716)	6.20 (0.323)	0.70 (0.008)	33.49 (0.201)	5.50 (0.052)	0.70 (0.004)	0.0044 (0.0008)	0.80 (0.011)
0.4	200	46.21 (1.787)	36.51 (0.134)	6.50 (0.038)	0.70 (0.002)	35.21 (0.566)	6.36 (0.268)	0.70 (0.003)	34.78 (0.572)	6.14 (0.271)	0.70 (0.003)	33.50 (0.123)	5.50 (0.032)	0.70 (0.0003)	0.0008 (0.0005)	0.79 (0.013)
0.4	500	46.17 (1.09)	36.51 (0.093)	6.50 (0.024)	0.70 (0.002)	35.29 (0.461)	6.40 (0.229)	0.70 (0.002)	34.72 (0.476)	6.11 (0.234)	0.70 (0.002)	33.50 (0.076)	5.50 (0.021)	0.70 (0.002)	0.0007 (0.0002)	0.80 (0.005)

The location of the simulated QTL is described by the map distances (in cM) from the first marker of the linkage group (100 cM long). The hypothesized *σ*^2 ^value is 0.0045 for *H*^2 ^= 0.10 and 0.00075 for *H*^2 ^= 0.4.

**Table 4:** The MLEs of the model parameters and the QTL position derived from 200 simulation replicates assuming no imprinting. The square root of the mean square errors (RMSEs) of the MLEs are given in parentheses.

			*Q*_*M*_*Q*_*P*_			*Q*_*M*_*q*_*P*_			*q*_*M*_*Q*_*P*_			*q*_*M*_*q*_*P*_			Residual	
							
*H*^2^	*n*	Position (cM)	*α*_1_ 36.5	*β*_1_ 6.5	*γ*_1_ 0.7	*α*_2_ 35	*β*_2_ 6	*γ*_2_ 0.7	*α*_3_ 35	*β*_3_ 6	*γ*_3_ 0.7	*α*_4_ 33.5	*β*_4_ 5.5	*γ*_4_ 0.7	*σ*^2^	*ρ* 0.8
0.1	200	45.24 (4.351)	36.74 (0.397)	6.53 (0.096)	0.698 (0.006)	35.09 (0.765)	5.99 (0.188)	0.70 (0.013)	35.35 (0.855)	6.09 (0.204)	0.70 (0.014)	33.71 (0.379)	5.54 (0.090)	0.70 (0.007)	0.004 (0.0009)	0.80 (0.02)
0.1	500	46.01 (2.196)	36.74 (0.327)	6.54 (0.070)	0.70 (0.004)	35.17 (0.567)	6.00 (0.138)	0.70 (0.012)	35.28 (0.640)	6.07 (0.160)	0.70 (0.012)	33.69 (0.273)	5.53 (0.058)	0.70 (0.004)	0.004 (0.0004)	0.80 (0.01)
0.4	200	46.07 (1.731)	36.56 (0.137)	6.51 (0.037)	0.70 (0.002)	34.99 (0.304)	6.00 (0.076)	0.70 (0.005)	35.11 (0318)	6.02 (0.076)	0.70 (0.005)	33.55 (0.127)	5.51 (0.033)	0.70 (0.003)	0.001 (0.0005)	0.80 (0.011)
0.4	500	46.17 (1.07)	36.56 (0.106)	6.51 (0.026)	0.70 (0.002)	35.03 (0.208)	6.00 (0.053)	0.70 (0.004)	35.07 (0.222)	6.01 (0.056)	0.70 (0.004)	33.54 (0.088)	5.51 (0.021)	0.70 (0.002)	0.0007 (0.0001)	0.80 (0.005)

The location of the simulated QTL is described by the map distances (in cM) from the first marker of the linkage group (100 cM long). The hypothesized *σ*^2 ^value is 0.0041 for *H*^2 ^= 0.10 and 0.0007 for *H*^2 ^= 0.4.

Under different simulation scenarios, another general trend is that the estimation for the genetic parameters of the two homozygotes performs better than that for the two reciprocal heterozygotes. For example, the RMSE of the growth parameter *a *for *Q*_*M*_*q*_*P *_is 0.765, while it decreases to 0.397 for genotype *Q*_*M*_*Q*_*P *_with fixed sample size 200 and heritability level 0.1 (Table 4). This is what we expected since partitioning the heterozygote into two parts may cause information loss. As the sample size or the heritability level increases, the RMSEs are greatly reduced for the two reciprocal heterozygous genotypes. For example, the RMSE (for parameter *a*) is reduced from 0.765 to 0.304 when the heritability level increases from 0.1 to 0.4 under fixed sample size 200 (Table 4). Overall, the QTL position estimation is reasonably good under different simulation scenarios, even though the precision is reduced a little with completely imprinted models (Tables 1 and 2), compared with the non-imprinting and partial imprinting models (Tables 3 and 4).

Table 1 also summarizes the results of comparison between the imprinting and non-imprinting models, in which the regular non-imprinting functional mapping model is indicated by "NI". Data are simulated assuming complete maternal imprinting, and are then subject to analysis using the imprinting (four QTL genotypes) and non-imprinting (three QTL genotypes) models. It can be seen that the non-imprinting model produces poorer estimation than the imprinting model. The RMSE is generally large when data are analyzed with the non-imprinting model, especially the mean parameters for the two reciprocal heterozygotes. We observed similar results under other imprinting mechanisms (e.g., partial or complete paternal imprinting) and the results are omitted. When data are simulated assuming no imprinting, the non-imprinting model, however, outperforms the imprinting model, in which the standard errors of the mean parameters fitted with the imprinting model are slightly higher than those fitted with the non-imprinting model (data not shown). Similar results were also obtained in our previous univariate imprinting analysis [22]. Therefore, caution is needed about the interpretation of the results. One should try both imprinting and non-imprinting models and report the union of QTLs that are shown in both analyses.

### A Case Study

We apply the developed model to a published data set [36] to show the utility of the approach. The data contain 502 F_2 _mice derived from two inbred strains differing greatly in body weight, the Large (LG/J) and Small (SM/J). Each F_2 _progeny was measured for its body mass at 10 equally spaced weeks starting at day 7 after birth. Ninety-six codominant markers were obtained with an average length of ~23 cM spanning the 19 autosomes. For more information about the data, readers are referred to the original paper [36].

**Figure 1:**
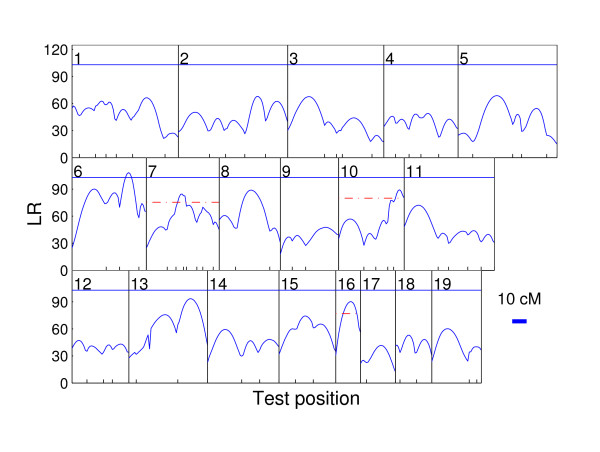
**Genomewide likelihood ratio profile plot**. The profiles of the log-likelihood ratios (LR) between the full and reduced (no QTL) model estimated from the functional imprinting model for body mass growth trajectories across chromosome 1 to 19 using the linkage map constructed from microsatellite markers [36]. The threshold value for claiming the existence of QTLs is given as the horizonal dotted line for the genome-wide level and dashed line for the chromosome-wide level. The genomic positions above the threshold line and corresponding to the peaks of the curves are the MLEs of the QTL positions. The positions of markers on the linkage groups [36] are indicated at ticks.

The sex-specific recombination rate is reconstructed on the basis of the marker data according to the average 1.25:1 recombination rate between female and male chromosomes [37]. The data were analyzed previously with a univariate imprinting model [12], in which three iQTL and one Mendelian QTL were detected. Considering the limitation of the univariate analysis as introduced in the background section, we apply the data to the newly developed functional mapping model. As clearly shown by the genomewide LR profile plot in Fig 1, the model detects four major QTLs. One QTL passes the genomewide threshold and is located on chromosome 6. The other three QTLs, located on chromosomes 7, 10 and 16, respectively, are only significant at the chromosome-wide level. In the plot, the solid curve corresponds to the likelihood ratio statistics at every testing position. The 5% significance threshold value for claiming the existence of QTLs at the genome-wide level is marked with the horizonal solid line based on permutation tests. The dashed line represents the 5% chromosome-wide threshold, which is obtained by only scanning the targeted chromosome.

Table 5 tabulates the estimated QTL positions, the marker intervals of the QTL belonging to each, the MLEs of curve parameters that specify the developmental pattern, and the asymptotic standard errors of the estimators (in parentheses). It can be seen that all parameters can be reasonably estimated with small sampling errors. The four QTLs detected are located between markers Nds5 and Mit15 on chromosome 6, between markers Nds1 and Mit148 on chromosome 7, between markers Mit133 and Mit14 on chromosome 10, and between markers Mit2 and Mit5 on chromosome 16. The data are also analyzed with the regular functional mapping approach and four QTLs are detected, among which only two QTLs, located on chromosomes 6 and 7, agree in both models. The other two, located on chromosomes 11 and 15, do not show significance with the imprinting analysis. The miss-detection of these two QTLs by the imprinting model may partially be due to the limitation of the imprinting approach as revealed by the simulation study.

**Table 5:** The QTL location, MLEs of the estimated parameters and their asymptotic standard errors in the parentheses with the AR(1) covariance structure.

		*Q*_*M*_*Q*_*P*_			*Q*_*M*_*q*_*P*_			*q*_*M*_*Q*_*P*_			*q*_*M*_*q*_*P*_			Residual	
						
CH*	QTL position	*α*_1_	*β*_1_	*γ*_1_	*α*_2_	*β*_2_	*γ*_2_	*α*_3_	*β*_3_	*γ*_3_	*α*_4_	*β*_4_	*γ*_4_	*ρ*	*σ*^2^
6(M)	*Nds*5-*Mit*15 (*Nds*5+12 cM)	36.53 (0.362)	6.47 (0.104)	0.67 (0.007)	33.09 (0.408)	7.01 (0.136)	0.67 (0.008)	36.11 (0.392)	5.87 (0.097)	0.66 (0.007)	33.36 (0.352)	5.90 (0.106)	0.66 (0.008)	0.77 (0.011)	0.012 (0.0005)
7(NI)	*Nds*1-*Mit*148 (*Nds*1+7.5 cM)	36.28 (0.651)	6.53 (0.098)	0.65 (0.006)	35.88 (0.434)	5.79 (0.153)	0.63 (0.009)	34.44 (0.357)	6.72 (0.091)	0.68 (0.007)	32.86 (0.317)	6.13 (0.086)	0.67 (0.006)	0.81 (0.009)	0.013 (0.0004)
10(M)	*Mit*133-*Mit*14 (*Mit*133+6.7 cM)	35.93 (0.335)	6.49 (0.094)	0.66 (0.006)	33.24 (0.436)	6.90 (0.139)	0.69 (0.009)	36.32 (0.398)	5.87 (0.097)	0.63 (0.007)	33.87 (0.329)	6.08 (0.090)	0.65 (0.006)	0.79 (0.008)	0.012 (0.0005)
16(M)	*Mit*2-*Mit*5 (*Mit*2+20 cM)	35.58 (0.369)	6.28 (0.103)	0.67 (0.007)	33.27 (0.439)	7.18 (0.145)	0.68 (0.009)	35.97 (0.441)	5.90 (0.106)	0.64 (0.008)	34.52 (0.367)	5.98 (0.099)	0.65 (0.007)	0.78 (0.013)	0.011 (0.0007)

*CH refers to chromosome, M refers to maternal imprinting, and NI refers to no imprinting.

**Figure 2:**
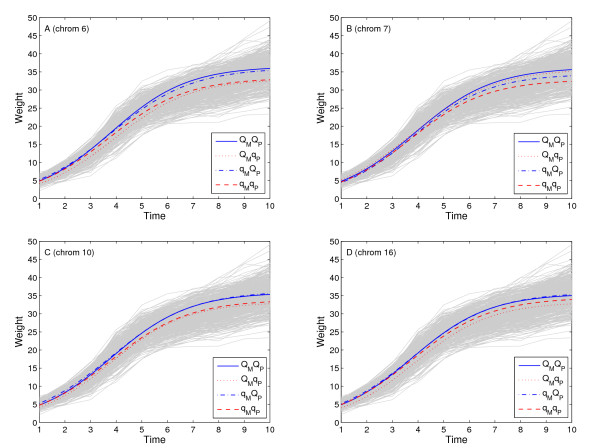
**Growth trajectory plot**. Four curves for the dynamic changes of mouse growth trajectory, each representing one of the four groups of genotypes, *Q*_*M*_*Q*_*P*_, *Q*_*M*_*q*_*P*_, *q*_*M*_*Q*_*P*_, and *q*_*M*_*q*_*P*_, at each of the four significant QTLs. Mouse growth trajectories for all observed individuals are indicated in gray background.

To dissect the imprinting property of the detected QTLs, we further conduct hypothesis tests based on Tests (11)–(13). Among the four QTLs detected, three show signs of imprinting, i.e., the ones located on chromosomes 6, 10 and 16. The one located on chromosome 7 shows no sign of imprinting. The developmental trajectories of the identified QTLs are plotted and are shown in Fig. 2 with the growth trajectories for all individuals indicated as gray in the background. The imprinting property of the four QTLs can also be inferred from the plot. For example, for the iQTL detected on chromosome 6, the solid and the dashed blue curves for QTL genotypes *Q*_*M*_*Q*_*P *_and *q*_*M*_*Q*_*P *_are almost merged together and the solid and dashed red curves for QTL genotypes *Q*_*M*_*q*_*P *_and *q*_*M*_*q*_*P *_are almost merged together. This implies that only the allele inherited from the paternal parent is expressed and the QTL is maternally imprinted. A similar result is obtained for this QTL with our previous univariate imprinting model [12]. A similar pattern can be seen for other two imprinted QTLs, while for the QTL detected on chromosome 7, four separate developmental curves can be clearly seen, which indicates that the QTL is not imprinted. The trajectory plots confirm the testing results based on Tests (12) and (13).

## Discussion

We all know that the diversity of offspring genes inherited from parents is due to the perturbation and reshuffling of parental genetic information during meiosis. Maintaining a functional expression balance between the paternal and maternal alleles is crucial for an organism's normal development. Breakage of the balance (e.g., the same allele expresses differently depending on its parental origin) often results in a phenomenon called genomic imprinting. In extreme cases, when one allele is completely silent, deletion of the functional allele may have serious consequences. For example, a deletion of the paternal copies of the imprinted SNRPN gene and necdin gene on chromosome 15 located in the region 15q11–13 may result in a genetic disorder called Prader-Willis syndrome [38].

Genomic imprinting has been ubiquitously observed in nature. The cause of imprinting has been thought to be related to DNA methylation in which certain parts of the DNA sequence are methylated and hence are silent in expression [39]. Although the functional mechanism of genomic imprinting is not totally clear, scientists thought that genomic imprinting has the one-generation dependence property. At the early stage of meiosis, the imprinting mark is erased and is subsequently reset at the end of meiosis, and imprinting is passed from one generation to another [40]. Offspring expression of an imprinted gene only depends on the allelic origin and is independent of the offspring sex. Therefore, any population in which the allelic parental origin can be traced can be used for mapping purposes theoretically.

Experimental line crossing has been widely used in QTL mapping studies. Several studies have been reported based on inbred line crosses for iQTL mapping [11-13]. These approaches are developed for univariate QTL analysis. Considering the functional dynamics of a gene, a powerful approach for understanding the genetic architecture of a dynamic trait would be to incorporate the dynamic feature of gene function into a mapping model. Statistical functional mapping, as revealed by empirical studies [20,22], shows its unique merits in mapping QTL underlying developmental character or reaction norm. To understand the genetic architecture of a dynamic imprinted trait, we have extended the functional mapping approach to map iQTL responsible for a dynamic imprinted trait. The model is a natural extension of our previous single trait imprinting model [12]. Simulation studies have shown the small sample properties of the approach under different simulation scenarios, considering the effect of sample size, heritability levels and different imprinting mechanisms.

As revealed by the real data analysis, the current functional iQTL mapping approach and our previous single trait iQTL mapping approach [12] agree on most significant QTLs, i.e., the ones from chromosomes 6,7 and 10. The imprinting property of these QTLs also agrees with the previous results, i.e., the QTLs on chromosome 6 and 10 are maternally imprinted and the QTL on chromosome 7 is not imprinted. However, the new model identifies one new iQTL located on chromosome 16. This iQTL is maternally imprinted. The one located on chromosome 15 detected by our previous single trait iQTL model does not show significance in the current analysis. Since the single trait analysis is based on the marginal distribution of the data at certain developmental stage, it may amplify the QTL effect by not adjusting the cumulative genetic effects up to time *t *[18]. Further experimental evidence is needed to confirm the results.

It is interesting to note that the detected iQTL for this data set are all maternally imprinted. The results, however, are not unusual, as explained by the parental genetic conflict theory [41]. Based on the theory, paternally derived alleles always trigger a favorable effect on the offspring's growth, whereas maternally derived alleles tend to trigger the opposite effect. As a result, imprinted genes that are preferentially expressed from the paternal alleles are enriched in genes that promote offspring growth. Therefore, for the growth trait in the current study, it is not surprising to see many maternally imprinted QTLs.

As a first attempt of its kind, we construct our functional iQTL mapping idea on a tractable one-QTL interval mapping framework. A one-QTL model does not consider the effects of background markers and is very limited to precisely elucidate the complex genetic architecture of a dynamic imprinted trait. The incorporation of ideas from more advanced mapping approaches such as composite interval mapping [42] and multiple interval mapping [43] can greatly enhance the utility of the developed method. More recently, Yang et al. [44] developed a composite functional mapping approach, which adopted a similar idea as the composite interval mapping [42] and shows improved features against the one-QTL functional mapping model. To make our work more useful in practice, modelling of multiple QTLs by composite or multiple interval mapping will be considered in future work.

The developed functional iQTL mapping is illustrated using an inbred F_2 _design. Since the allele parental origin for the two reciprocal heterozygotes is not distinguishable, we incorporate the sex specific recombination rate information to discern the difference in distribution of the two reciprocal heterozygotes. We assume an average 1.25:1 female-to-male recombination rate in mouse to illustrate our method. Additional simulations are conducted by varying the recombination ratio and similar results are obtained (data not shown). The sex specific recombination rates are commonly observed in nature. For example, averaged over the entire genome, the female-to-male recombination rate is 1.6:1 in human [45], 1.4:1 in dog [46], 1.4:1 in pig [47]. However, we also expect local variation in sex specific recombination rate. For example, Marklund et al. [47] reported that the chromosome specific female-to-male recombination rate varies from 2.2:1 (Pig chromosome 5) to 1:1 (Pig chromosome 1), an approximately two-fold difference in female-to-male recombination rates. This kind of local variation in sex specific recombination rate may affect the analysis results, and concomitantly the inference of genomic imprinting. The use of a sex-specific linkage map would greatly improve the inference of a local imprinting property of a QTL. With the availability of sex specific linkage information in experimental species, we expect the method be more robust and to provide more informative results. Meanwhile, owning to the limited information about the sex specific recombination rate in other species, this may limit the application of the developed approach to other populations. An alternative genetic design that can trace the allelic parental origin using experimental line crosses is the backcross design. Statistical approaches focusing on the backcross design have been developed for iQTL mapping in univariate trait analysis [11,13]. The idea can be extended to functional iQTL mapping with little modification.

Functional mapping and its extension like the one presented in this article provide a stimulating way to map complex biological processes by incorporating curve fitting into a mapping framework. Regardless of the limitations mentioned above, the integration of imprinting information into the functional mapping framework provides a testable quantitative platform for understanding the genetic basis of imprinted genes accounting for quantitative variation of a dynamic trait. The incorporation of more flexible mean function modelling approaches, such as non-parametric regression [48], would greatly enhance the flexibility of the current approach.

## Competing interests

The author(s) declare that they have no competing interests.

## Authors' contributions

YC conceived the idea, developed the model, performed the analysis, and drafted the manuscript. SL and GL assisted in the analysis and preparation of the manuscript.
